# Building an Enhanced Vocabulary of the Robot Environment with a Ceiling Pointing Camera

**DOI:** 10.3390/s16040493

**Published:** 2016-04-07

**Authors:** Alejandro Rituerto, Henrik Andreasson, Ana C. Murillo, Achim Lilienthal, José Jesús Guerrero

**Affiliations:** 1 Instituto de Investigación en Ingeniería de Aragón, Deptartmento de Informática e Ingeniería de Sistemas, University of Zaragoza, Zaragoza 50018, Spain; acm@unizar.es (A.C.M.); jguerrer@unizar.es (J.J.G.); 2 Centre for Applied Autonomous Sensor Systems, Deptartment of Technology, Örebro University, Örebro SE-70182, Sweden; henrik.andreasson@oru.se (H.A.); achim.lilienthal@oru.se (A.L.)

**Keywords:** visual vocabulary, computer vision, bag of words, robotics, place recognition, environment description

## Abstract

Mobile robots are of great help for automatic monitoring tasks in different environments. One of the first tasks that needs to be addressed when creating these kinds of robotic systems is modeling the robot environment. This work proposes a pipeline to build an enhanced visual model of a robot environment indoors. Vision based recognition approaches frequently use quantized feature spaces, commonly known as Bag of Words (BoW) or vocabulary representations. A drawback using standard BoW approaches is that semantic information is not considered as a criteria to create the visual words. To solve this challenging task, this paper studies how to leverage the standard vocabulary construction process to obtain a more meaningful visual vocabulary of the robot work environment using image sequences. We take advantage of spatio-temporal constraints and prior knowledge about the position of the camera. The key contribution of our work is the definition of a new pipeline to create a model of the environment. This pipeline incorporates (1) tracking information to the process of vocabulary construction and (2) geometric cues to the appearance descriptors. Motivated by long term robotic applications, such as the aforementioned monitoring tasks, we focus on a configuration where the robot camera points to the ceiling, which captures more stable regions of the environment. The experimental validation shows how our vocabulary models the environment in more detail than standard vocabulary approaches, without loss of recognition performance. We show different robotic tasks that could benefit of the use of our visual vocabulary approach, such as place recognition or object discovery. For this validation, we use our publicly available data-set.

## 1. Introduction

Bag of Words (BoW) approaches are a common way to represent images based on a quantized feature space. They are broadly used in visual recognition problems, such as object or place recognition and image retrieval [1,2,3,4,5,6]. These techniques create a catalog of image features or words and describe each image as a vector of occurrence counts of these words.

A typical drawback using standard BoW approaches is that semantic information is usually neglected when grouping the visual features into the clusters or visual words. In a general setting, including conceptual information is challenging since no assumption can be made about the type and meaning of the visual features that may appear. However, many applications could benefit from including semantic content in the vocabulary to achieve their goals. Our objective is to build an improved vocabulary that provides a more meaningful model for applications using BoW techniques. We focus on mobile robotic applications which imply long term operations indoors, in environments such as a warehouse [7] or a museum [8].

Our approach is built after two intuitive hypotheses. First, robotic platforms provide sequential information (a sequence of images) including tracking information, while building the vocabulary helps to cluster the different appearances of a scene element. Second, cameras in robotic platforms have a fixed location to the robot, respectively. We can find spatial restrictions and make reasonable assumptions about the location of elements of the environment (e.g., the lamps are on the ceiling, the posters on the wall, ...). We use simple geometric information together with the image appearance to cluster the environment elements.

As done previously in multiple robotics applications [9,10], we benefit from having a camera pointing to the ceiling. Such camera configuration improves the operation over long periods of time, something crucial to address indoor monitoring applications with autonomous systems. Upper parts of indoor scenes are typically less dynamic than the rest of the scene and provide a more robust visual model. In this setting, we expect the environment to include a small number of repeatable elements (different kinds of lamps or windows) and a few elements which are rather unique (exit signs, posters or labels), most of them with fixed locations within the scene. We assume that these elements present a restricted set of appearances from a few points of view that are reachable by our robot.

**Figure 1:**
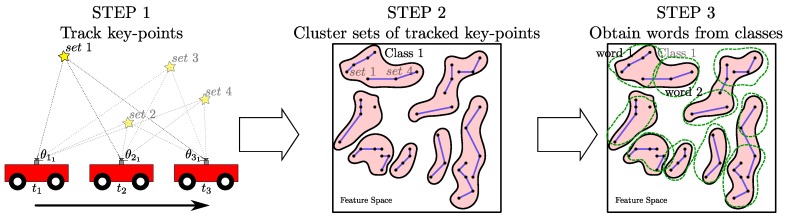
Diagram of our novel vocabulary construction method. During the acquisition, extracted image key-points are tracked. All the image appearances and altitude angle values of these key-points are stored and grouped together under the same set number. These sets of tracked key-points are later clustered into classes, with the elements in each class representing the same element of the environment. Finally, words are extracted running a clustering for each of the obtained classes.

Our approach consists of a novel hierarchical process (summarized in Figure 1). It creates a visual vocabulary with richer information about the environment than standard BoW methods. Instead of computing the visual words directly from the acquired data, we propose obtaining an initial grouping into classes containing similar sets of tracked key-points. Then, standard vocabularies are computed in each of those classes.

The main differences and contributions of our approach with regard to prior work are the following:

- We leverage the visual vocabulary for a more meaningful representation of a given working environment;
- We propose a novel way to include spatio-temporal information in the vocabulary construction process: thanks to feature tracking, our approach automatically groups the different appearances of scene elements;
- Additionally, we propose including each key-point altitude value in the key-point descriptors to encode the different viewpoints for the scene elements.

Our experimental section demonstrates how the presented approach builds a visual model that provides higher representativity of the environment. At the same time, the created vocabulary maintains the same performance for place recognition as a standard vocabulary.

## 2. Related Work

How to acquire a representative visual model of the environment is a problem that has been studied for a long time. In particular, acquiring models of the environment for long term operation is a subject of great interest, since it provides intelligent systems with higher autonomy [11,12]. Additionally, enhancing those models with semantic information is a key element for human–computer interaction [13,14,15,16,17].

Seeking for robust models across time, indoor vision based robotic tasks have taken advantage of using visual ceiling features [9,10]. Elements on ceilings are usually more stable over time than those in floors or walls, where dynamic scene elements often appear. Recent results revisited this idea for indoor and industrial oriented robotic applications [18,19], where long term operation is required.

Robustness during long term operations is an issue of great importance when designing autonomous systems for indoor environment monitoring and surveillance [20,21,22]. In such applications, the robot needs to reliably localize itself in a known environment and have some semantic information that allows the system to measure parameters of interest or discover certain events, such as the presence or absence of common objects or changes in the environment infrastructure (potential changes may be due to broken components, leaks, *etc.*).

One of the most popular approaches to build visual models is the Bag of Words (BoW) representation. It is based on a quantization of the image feature descriptor space into several groups (visual words) that compose the visual vocabulary. BoW based approaches are very popular in various recognition tasks due to their good performance despite the simplified representation. Authors of [23] perform a survey of current visual topological mapping methods concluding that BoW approaches are better than global descriptor or local feature based approaches for large scale operation. We find good results on recognition at large scale achieved partly thanks to BoW image representation, such as appearance based localization and mapping in large scenarios [5]; object retrieval approaches [2], or event detection methods [24].

In a seminal work from Sivic *et al.* [1], authors propose to use tracked features to build a visual vocabulary because they are more likely to be stable features. Inspired by these ideas, we use key-point tracking not only to find stable features but to discover the different appearances of the environment elements from a different viewpoint. In [25], authors extract the different appearances of the elements from a structure-from-motion point cloud of the environment. Given the different viewpoints, different image descriptors, of a 3D point, the number of appearances is reduced using mean-shift clustering. Using these set of appearances, authors localize new images of the environment in the created 3D map. Work in [26] makes use of feature tracks that in this case are clusters of image patches of similar appearance. Authors infer relationships between visual words given these feature tracks and learn a new metric to compare image features. This metric estimates the probability of observing a word of the vocabulary given an observed word in the query image. Probability will be high if multiple feature tracks include features assigned to both words and low otherwise. Contrary to our proposal that tracks the key-points of the scene to detect their appearance, these works group features by matching their descriptors.

In parallel with the growing popularity of vocabulary based recognition approaches, we find research results analyzing their drawbacks [27,28], such as the loss of discriminative information or under-representation of descriptor space regions with low population. Therefore, we also find approaches trying to overcome some of these issues, as well as augmenting the model semantic information. For example, [29] presents a novel framework for object category recognition that unifies visual codebook generation with classifier training. Traditional visual vocabulary learning and weighting is performed independently, and authors of [30] present Joint-ViVo, a method where words and their weights are learned jointly. In [31], authors improve the BoW creation by selecting informative words depending on a saliency value computed in the images. The approach described in [32] proposes a supervised learning algorithm which generalizes the *k*-means algorithm by allowing soft assignments and exploits supervised information to improve the discriminative power of the vocabulary. Work in [33] studies the problem of large scale image retrieval by developing a new class of bag-of-features to encode geometric information of objects within an image. Authors of [34] present a framework to integrate semantic information from Flickr labels for supervised vocabulary construction. The work in [14] propose a framework to solve the missing correspondences between low-level multimedia data and high-level semantics. This framework can be used to link the elements in a visual vocabulary with the elements of a semantic vocabulary.

One of our intuitions is that geometric and spatial restrictions on the scene are important cues to improve our visual model. Therefore, we include spatial information in the form of the key-points altitude as part of our key-points descriptor. Previous work has already proved the advantages of using geometric cues in visual models. For example, the work in [35] presents the Geometric min-Hashing. This method uses semi-local geometric information to create and increase the discriminative power of the hash keys, improving their visual model. In [36], spatial pyramids are used to characterize the spatial arrangement of words in the image. The methods in [37,38] include the relative spatial configuration of the words to improve the vocabulary. The first work introduces a bag of spatio-visual words representation (BoSVW) obtained by clustering of visual words correlogram. The second work uses spatial arrangement of visual words (WSA) for image retrieval and classification outperforming spatial pyramids in the retrieval scenario. In [39], angle and scale of image descriptors are used to improve image matching. Authors weigh the matching score of a query and a database image by verifying the consistency of angle and scale of all the matched words between the images.

Finally, there is another important group of related work regarding unsupervised learning for object or feature discovery. Unsupervised learning has been used to discover the distinctive architecture elements of certain areas [40], representative views of an object [41], object models [42] or the appearance of objects and their image segmentation [43]. Closer to our approach, the work in [44] finds a set of discriminative and representative image patches by using an iterative process of clustering and training. In [45], authors construct a compact and discriminative semantic visual vocabulary using diffusion maps and quantized mid-level features.

## 3. Enhanced Vocabulary Construction

This section details our approach to build an enhanced vocabulary of the environment traversed by a mobile camera. Steps are summarized in Figure 1: (1) feature detection and tracking that groups the features into sets of tracked key-points; (2) clustering sets of similar appearance into classes; (3) obtaining the visual words from each class.

### 3.1. Key-Point Detection and Tracking

We detect key-points in the scene and track them using the Lucas–Kanade tracker. For each frame, we compute an image descriptor around each key-point location. For both key-point detection and image descriptor computation, we use SURF (Speed-Up Robust Features) [46]. Note that the appearance of key-points is likely to change from frame to frame while they are tracked. We can exploit the knowledge of these appearance variations being generated by the same entity thanks to the tracking. Due to the camera configuration, pointing towards the ceiling, the altitude angle encodes the point of view change that produces the appearance change of the scene point. The tracked image descriptors are stored, together with the altitude *θ* of the corresponding location, as shown in Equation (1).

As a result of this step, our approach has identified *m* sets of tracked key-points, where each set, seti, contains $n_{i}$ image descriptors and altitude angles corresponding to the same tracked scene point from different points of view. $X_{j_{i}}$ denotes the *j*th descriptor and altitude angle pair included in seti:

(1) $$set\;i=\{X_{j_{i}}=\left[desc_{j_{i}},\;\theta_{j_{i}}\right]\}$$

with *j* in $\left[1\ldots n_{i}\right]$ and *i* in [1…m].

Different sets could contain features that belong to the same scene element, e.g., scene points tracked at different time while revisiting the same location or points corresponding to repeated objects in the environment. The following step of our approach groups sets that are likely to have been produced by the same object or element.

### 3.2. Clustering Sets of Tracked Key-Points

To cluster the sets of tracked key-points, we have evaluated two clustering methods: Hierarchical Clustering and DBSCAN (Density-Based Spatial Clustering of Applications with Noise). Both methods can work based on a similarity measure between sets.

#### 3.2.1. Similarity Measure

The similarity measure described next estimates how likely two key-point sets are to correspond to the same scene element. The altitude value encodes the relative position of the scene point with respect to the camera. We assume that the appearance of a scene point from the same viewpoint is the same, but different scene points can look similar from different positions. We use the altitude difference to penalize these cases.

The distance between two features $X_{i}=\left[desc_{i},\;\theta_{i}\right]$ and $X_{j}=\left[desc_{j},\;\theta_{j}\right]$, is computed using:

(2) $$d\left(X_{i},X_{j}\right)=||desc_{i},desc_{j}\left||f(|\right|\theta_{i},\theta_{j}\left||\right)$$

where $||desc_{i},desc_{j}\left|\right|$ is the Euclidean distance between the appearance descriptors and f(x) is the penalization due to the altitude difference between both features obtained as follows:

(3) f(x)=(1+aexp(bexp(cx)))

where *a*, *b* and *c* are the parameters that define the shape of the penalization.

**Figure 2:**
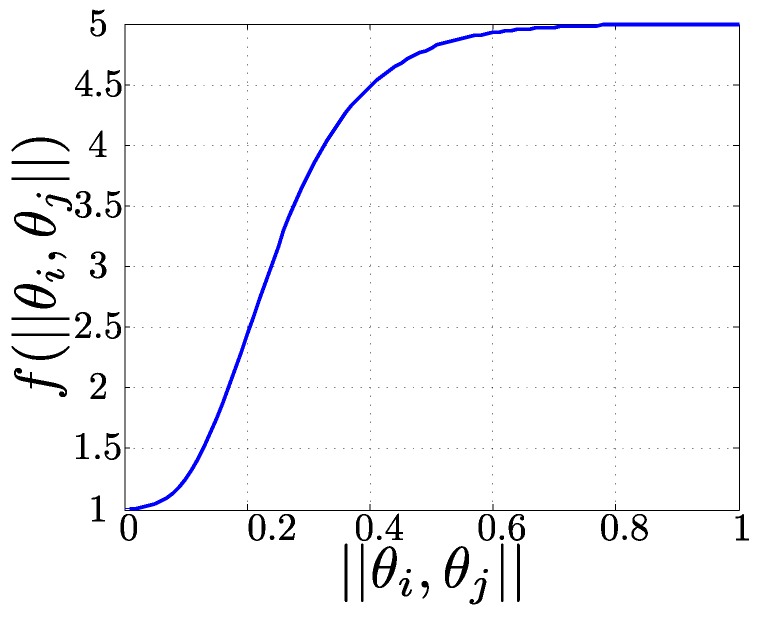
Penalization Equation (3): a=4, b=-7.5 and c=-10.

The penalization function is shown in Figure 2. It grows rapidly and continuously after a small difference of altitudes (0.1 rad), and the maximum penalization is used for values above 0.8 rad. Parameters *a*, *b* and *c* have been selected to accomplish these requirements. Other functions with the same properties are also valid. The selected function penalizes the matching of key-points observed from different altitude angles but does not modifies the matching other rotations of the key-points. In such cases, the rotation invariance of the chosen SURF descriptor allows for recognizing the key-point.

To compare two sets of tracked key-points, we have to compare all the features of one set with all the features from the other set. Each feature of seti is compared with all the features of setj, the minimum distance value is selected, and the mean of all these minimum values is considered as the distance between sets:

(4) $$D\left(set\;i,set\;j\right)=\underset{X_{k}\in set\;i}{mean}\left(\min_{X_{l}\in set\;j}\left(d\left(X_{k},X_{l}\right)\right)\right)$$

where D(seti,setj) is the distance between two different sets, seti and setj, and $X_{k}$ and $X_{l}$ are features included in these sets, respectively.

#### 3.2.2. Clustering Approaches

We consider two common clustering approaches that build on a similarity measure between the elements to be clustered.

- Hierarchical Clustering.
  We have implemented a clustering method based on the agglomerative Hierarchical Clustering [47], where each element starts as a cluster and each cluster is paired with its most similar cluster as we move up in the hierarchy. Our hypothesis is that elements in the same cluster are probably observations originated from the same object or scene point. We modify the standard Hierarchical Clustering by defining threshold $th_{S}$ to avoid merging too dissimilar clusters (clusters are not paired if their distance is above this threshold). As a result of this modification of the standard Hierarchical Clustering, key-points sets far from any other set are not paired and compose a singleton cluster. In this method, new clusters are created on every iteration, so new distances have to be computed. We adopt the Unweighted Pair Group Method with Arithmetic Mean [48] approach, where the distance between two clusters is the mean of the distances between the sets of tracked key-points included in each cluster.
  Hierarchical Clustering is conceptually simple, that means it is easy to implement and modify. Additionally, it outputs a hierarchy of clusters, a structure more informative than flat clustering technique results. The drawback is its complexity, $O(n^{3})$ in the general case, what makes it too slow for big data-sets.
- DBSCAN (Density-Based Spatial Clustering of Applications with Noise).
  DBSCAN [49] is a density-based clustering algorithm that uses a estimated density distribution of corresponding nodes to find clusters in the data. This algorithm is based in the notion of density reachability: Two elements, *q* and *p*, are directly density reachable if their distance is not bigger than *ε*. *q* is called density-reachable from *p* if there is a sequence of elements, $p_{1}\ldots p_{n}$ with $p_{1}=p$ and $p_{n}=q$, where each $p_{i+1}$ is directly density-reachable from $p_{i}$. With these definitions, a cluster is a subset of elements mutually density-connected. To handle the noise, this method defines the parameter minPts, the minimum number of elements required to create a cluster. Subsets of density-connected elements with less than minPts elements are considered as noise.
  DBSCAN is a widely used clustering technique. We use the DBSCAN implementation included in ELKI open source data mining software [50]. The complexity of this method is lower than for Hierarchical Clustering, $O(n^{2})$ for the basic form of the algorithm, so it is faster and more appropriate for big data-sets.

The results of this clustering step can be semantically understood as follows:

- The obtained clusters represent common scene points and include their possible appearances according to the different viewpoints under which the scene elements were observed.
- Non paired or noisy sets are unique scene points. These sets are dissimilar to the rest of sets but may be highly representative of the locations where they appear. These unique sets are clustered together after running the clustering step. The created cluster does not represent any common scene points, but includes unique key-points representative of a location. Unique sets are clusters with just one set of tracked key-points when using Hierarchical Clustering. When running DBSCAN with minPts=2, these unique sets are considered as noise.

### 3.3. Vocabulary Construction

Our last step to obtain the visual model consists of building a vocabulary for each class resulting from previous step. The whole vocabulary is composed by a fixed number of words *K*. We assign a number of words $k_{i}$ to each class *i*. $k_{i}$ is proportional to the number of features included in class *i*:

(5) $$k_{i}=\left\lceil K\;\frac{\#\;features\;\in class\;i}{\#\;total\;features}\right\rceil$$

*K*-means clustering algorithm is run with the elements within each class. Differently from previous steps, *k*-means is run using only the appearance descriptors of the features in that class. Each word gets a representative appearance descriptor from *k*-means. We add an altitude value computed as the average altitude of all features assigned to that word.

In the resulting vocabulary, larger classes are represented by more words than small ones. The class including non paired sets, which is usually big, will receive a large amount of words, which guarantee that we account for these marginal and unique scene elements.

### 3.4. Assigning Words to a New Feature Using the Created BoW

To classify a new feature, $X_{new}$, into the discovered classes, it is compared with the words included in the vocabulary using the distance described in Equation (2). We assign the corresponding word *i* according to the nearest neighbor, but only if that distance is below a matching threshold, $th_{M}$.

(6) $$i=\underset{i\in [1,k]}{\arg\min}\left(d\left(X_{new},X_{i}\right)|d\left(X_{new},X_{i}\right)<th_{M}\right)$$

By using this threshold, we model the fact that a new feature could not belong to any of the modeled classes.

## 4. Analysis of the Performance of the Hierarchical Vocabulary

We have evaluated all steps of our approach and the performance and properties of the obtained visual vocabulary.

### 4.1. Experimental Settings

#### 4.1.1. Data-Sets

**Figure 3:**
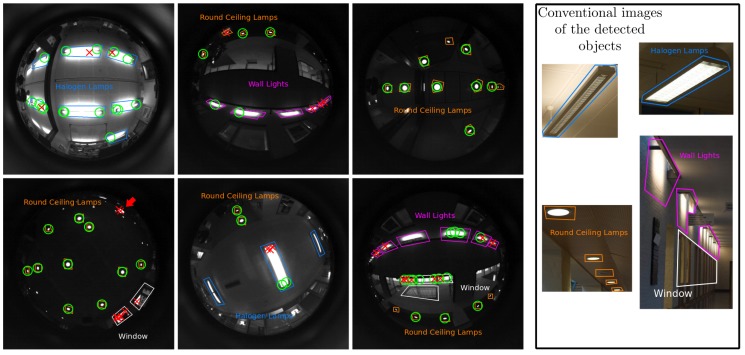
Examples of objects discovered in the data-set. Green circles mark correct assignments and red crosses show incorrect ones. The colored shapes show the labeled areas: round Ceiling Lamps (**orange**); Halogen lamps (**blue**); wall Lights (**magenta**); windows (**white**). Matching threshold is set to 0.15. For clarity, some conventional images of the objects in the environment are shown on the right of the figure. (Best seen in color).

We validate our method with a data-set acquired from a robotic platform at the AASS laboratories and offices in Örebro University, Sweden. It includes two image sequences of two different trajectories around the same environment: 158.5 m (1879 frames) and 164 m (2142 frames). They were acquired at different days, in the same environment and follow different trajectories. The acquisition was performed at 30 frames per second, and the images have a resolution of 768×768 pixels. As explained previously, the camera has been set pointing to the ceiling, therefore the objects that appear are mostly light sources, windows and signs. Figure 3 shows some sample images of this data-set, which is available online [51].

A second data-set is used for qualitative evaluation of the method. This sequence has been acquired in a trajectory of about 10 m on a different indoor environment, traversing a corridor. The purpose of this sequence is to further analyze the correspondence between classes and real objects in a different scenario than the main data-set used.

#### 4.1.2. Performance Measurements

The proposed method is evaluated attending to three different criteria:

- *Accuracy:* it evaluates the accuracy of the vocabulary to classify new features into the discovered classes. Total, $A_{Total}$, and average class accuracy, $A_{Average}$, of the classification are respectively computed as:
  (7) $$A_{Total}=100\frac{\#\;correct\;class}{\#\;test\;features}$$
  (8) $$A_{Average}=\underset{\forall i}{mean}\left(100\frac{\#\;correct\;class_{i}}{\#\;test\;features_{i}}\right)$$
  where the *i* index represents the classes.
- *Normalized inverse pixel deviation:* it evaluates the similarity of the key-points patches included in each class. This quality measurement is based on the standard deviation of the image patches of features that have been clustered together. Given class *i*, we define the class pixel deviation, $S_{i}$, as the mean of the standard deviation of the gray level of every pixel of the features patches included in class *i*:
  (9) $$S_{i}=\underset{\forall (x,y)}{mean}\left(\underset{\forall j\in i}{std}\left(I_{j}\left(x,y\right)\right)\right)$$
  where j∈i represents all the patches of the features included in the class *i*, $I_{j}\left(x,y\right)$ is the gray level of pixel (x,y) from the patch of feature *j*, (x,y) values are limited to the size of the patches (32×32 pixels in our case) and std() is the standard deviation.
  We define the normalized inverse pixel deviation for each class, $S_{i}^{\prime }$:
  (10) $$S_{i}^{\prime }=1-\frac{S_{i}}{S_{max}}$$
  where $S_{max}$ is the maximum pixel deviation. More meaningful classes will have higher $S_{i}^{\prime }$ values.
- *Intra-class distance:* it evaluates the similarity between all the sets of key-points included in each class. Distance between all the sets of key-points is computed using Equation (4). The intra-class distance is the mean of these distances. Lower values of this distance mean more compact clusters, where the sets grouped are more similar.

Normalized inverse pixel deviation and intra-class distance both evaluate how similar are the elements grouped under the same class label. However, the first one computes distances between key-point patches, just the key-points appearance, while the latter computes distances between sets of key-points using tracking and viewpoint information together with the SURF descriptors.

#### 4.1.3. Comparison with *k*-Means Vocabulary

**Figure 4:**
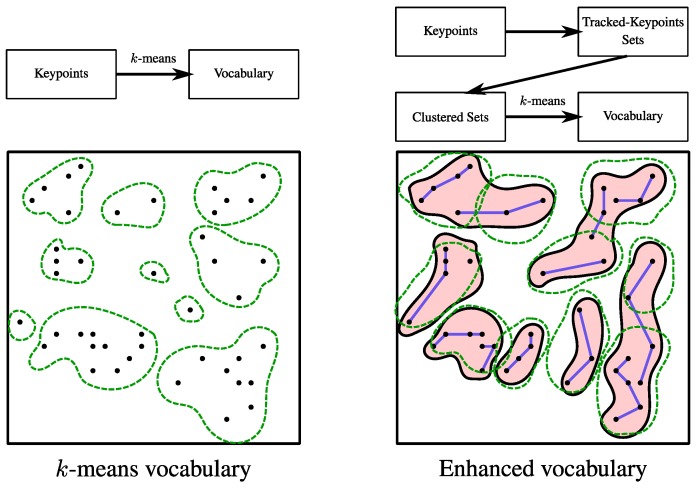
Differences between standard *k*-means vocabulary and our hierarchical vocabulary building process. *k*-means clusters directly in the feature descriptor space. Our enhanced vocabulary first groups by tracking, then clusters tracked key-point sets into classes, and finally clusters with a k-means within each of the found classes to create the final visual words.

Next, experiments compare the properties of our proposed vocabulary with those of the standard *k*-means vocabulary. We have chosen this technique as baseline for the visual vocabularies. Standard *k*-means has shown good performance in plenty of computer vision applications, but one of its drawbacks is that not semantic information is considered when building the words. In contrast, the hierarchical vocabulary presented in this work groups in the same class key-points that are likely to come from the same scene element. Figure 4 shows the basic differences between both approaches.

### 4.2. Analysis of the Clustering of Tracked Key-Points Sets

**Figure 5:**
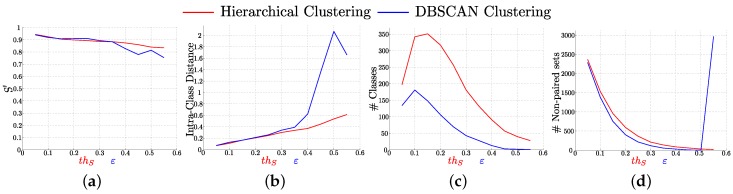
Comparison of the clustering results for both studied methods: Hierarchical Clustering and DBSCAN Clustering. Evaluation of the normalized inverse pixel deviation (**a**); intra-class distance (**b**); number of created classes (**c**) and number of non paired classes (**d**) for both Hierarchical Clustering (**red**) and DBSCAN (**blue**) clustering. $th_{S}$ and *ε* are equivalent parameters for both clustering methods respectively. (Best seen in color).

Figure 5 shows the evolution of the main parameters of the clustering results for different configurations of the two evaluated clustering algorithms. We can observe how the intra-class distance and the number of non paired sets have similar behavior for values of $th_{S}$ and *ε* between 0.05 and 0.35. However, DBSCAN creates less clusters than Hierarchical Clustering. In DBSCAN, two elements can be clustered together if they are density reachable, even if their distance is high. In contrast, in our modified Hierarchical Clustering, to pair two elements the distance between these elements must be lower than $th_{S}$. The requirement to cluster elements is more relaxed for DBSCAN, so more sets are clustered together and less clusters are created than with Hierarchical Clustering. For *ε* higher than 0.35, very few clusters are created with DBSCAN. This means that dissimilar sets are clustered together affecting the quality of the results as can be seen in the evolution of $S^{\prime }$ and the intra-class distance. Figure 5d shows a peak on the number of non-paired sets when $th_{S}>0.5$. This is caused because there are no sets which similarity distance with other set is below that threshold. As a result of this, all sets are grouped together in the non-paired cluster.

### 4.3. Influence of $th_{S}$, ε and K Parameters in the Performance of the Resulting Vocabulary

**Figure 6:**
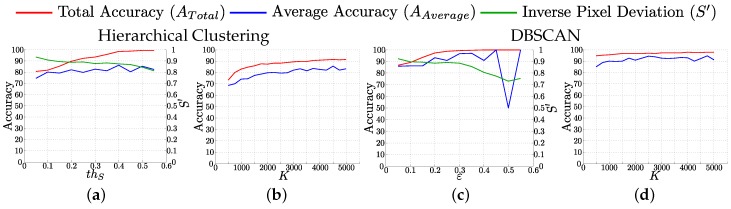
Influence of parameters, $th_{S}$, *ε* and *K* in the performance of the vocabulary. We show the evolution of total (**red**) and average (**blue**) accuracy and normalized inverse pixel deviation (**green**) for $th_{S}$ (**a**) and *ε* (**c**) with K=3000 and for *K*; (**b**) and (**d**), for $th_{S}$ and *ε* equal to 0.1.

For this analysis, we randomly split the sequences and use 70% as training, and 30% as test data. The clustering of sets of tracked key-points into classes is performed on the whole sequence to define a ground truth class assignment for both train and test data. The vocabulary is built using only the training data and the next results correspond to the classification of the test data into vocabulary classes. Figure 6 shows the Total and Average accuracy of the classification and the Normalized inverse pixel deviation.

For Hierarchical Clustering, $A_{Total}$ grows with $th_{S}$, while $A_{Average}$ remains constant. This means that larger classes get even larger by clustering dissimilar sets of tracked key-points. $A_{Total}$ increases due to the good performance of these large classes, but $A_{Average}$ remains constant due to the poor performance of the small classes. As expected, the quality of the classes, represented by $S^{\prime }$, decreases when $th_{S}$ grows.

For DBSCAN clustering, we can observe how both, $A_{Total}$ and $A_{Average}$, grow with *ε* while $S^{\prime }$ decreases. The high values observed for $A_{Total}$, are effect of the low number of clusters created. The chance of classifying correctly a key-point when most of the features are part of the same cluster is very high. $A_{Total}$ and $A_{Average}$ remain almost constant when *k* grows. Those values are in all cases higher than for Hierarchical Clustering because the first class created by DBSCAN clustering is too big, as we show later in Section 4.5. Most of the test key-points are classified under that class, so the accuracy is high. However, this class is compounded by dissimilar sets of tracked key-points. High values of *K* produce large vocabularies that better fit the feature space. However, one of the goals of using vocabularies is to reduce the number of elements included in the model, so small *K* values are preferred.

### 4.4. Influence of the Robot Motion in the Detection of Key-Point Classes

**Figure 7:**
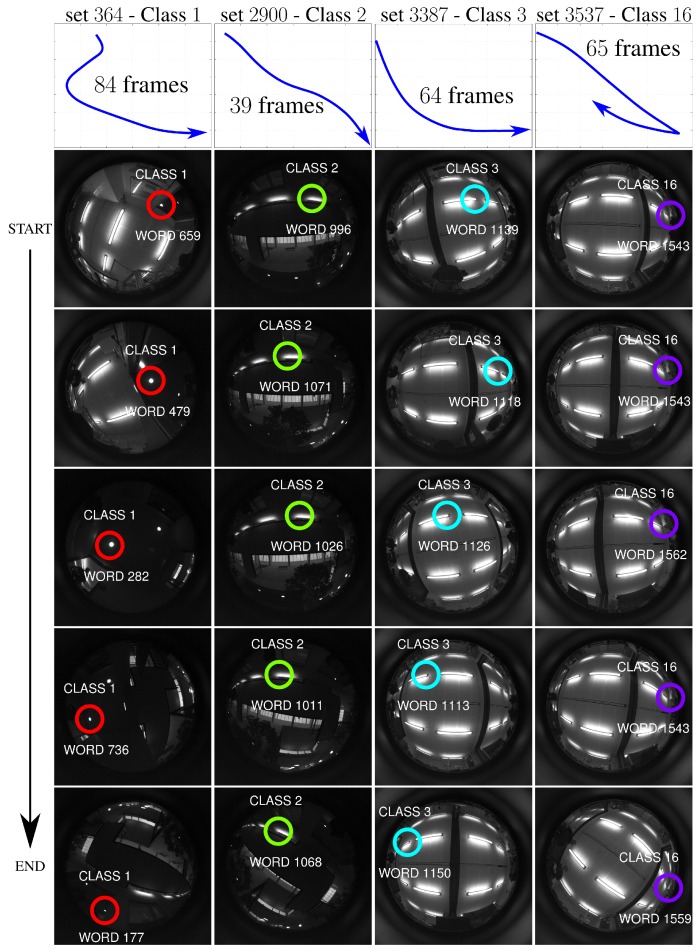
Key-point classification into words and classes as the robot moves. The plots in the first row represent the trajectories followed by the robot while acquiring the frames. The trajectories include rotation, translation and combinations of both. Each column shows how the key-points of a set of key-points are individually classified as being of the same class. All the key-points of a set correspond to the same element of the scene that has been tracked. (Best seen in color).

This experiment analyzes the suitability and correctness of our vocabulary to model the environment. We classify key-points from a test sequence not used to build the vocabulary into the classes discovered by our approach. Figure 7 shows examples of key-points classified along different test sub-sequences. Each column of the figure shows key-points detected in those frames together with the word and corresponding class assigned to that key-point. Note that as the key-point appearance and position varies, it is assigned to different words but is still classified under the same class. This validation demonstrates for a given scene element, *i.e.*, one of our classes, how the different words encode the element appearances and viewpoints.

### 4.5. Analysis of the Object Information Included in the Vocabulary

One interesting property of our method is that the classes created by clustering sets of tracked key-points are related to scene elements of the environment. We want to analyze if these discovered classes actually correspond to objects or parts of the environment (note that every step of our method is unsupervised, so we do not have any concept name associated with any of the classes).

For this experiment, we use both sequences, one for obtaining the vocabulary and the second one for testing. In both sequences, we have labeled manually 10% of the images with bounding boxes around the four most repeated objects in the environment: Halogen Lamp, Round Ceiling Lamp, Wall Light and Window (see Figure 3). Since the camera is pointing to the ceiling, the objects that appear are mainly different kinds of lamps and windows.

#### 4.5.1. Relationship between Words and Classes with the Environment Objects

First, we analyze how our proposal creates a visual vocabulary where the classes (and words subsequently) are related with the environment objects. A quantitative analysis of the relation between scene objects and the classes created by our vocabulary can be seen in Table 1. It shows the normalized entropy values for the classification of the objects into classes and words. Normalized entropy measures the unpredictability of a random variable. In our case, the random variable is the classification of a key-point generated by a scene element into words or classes. If the key-points of an object are always classified in the same word, the entropy will be zero. However, if object key-points are assigned into a lot of different words, the entropy value will be high. To compare the entropy of different vocabularies, we use the Normalized Entropy:

(11) $$Normalized\;Entropy=\frac{\left(-\sum_{i=1}^{n}f_{i}\log_{2}\left(f_{i}\right)\right)}{\log_{2}\left(n\right)}$$

where, for each object, $f_{i}$ is the proportion of occurrences of that object in the class or word *i*, and *n* is the number of classes or words.

Analyzing the classification at word level, the three vocabularies have a similar normalized entropy value (about 0.48). However, looking at the values for the classification into classes, the values are much lower (0.275 and 0.394 for our approach with DBSCAN and with Hierarchical Clustering, respectively). Using a standard *k*-means vocabulary (c), there is no relation between words and scene objects and words generated from the same objects are not related in any way.

#### 4.5.2. Classes Representing Objects

The following experiments show how the relation between the classes created by our approach and the objects of the environment can be used to detect the different object occurrences.

First, we define the concept of representative classes. A class is representative of an object if most of the class key-points are also key-points of that object:

(12) $$100\frac{\#\;class_{i}\;\text{key-points}\;\mathrm{in}\;object_{j}}{\#\;class_{i}\;\text{key-points}\;}\geq th_{R}$$

where $th_{R}$ is the representativity threshold which models how unique a class needs to be to be representative of an object.

**Figure 8:**
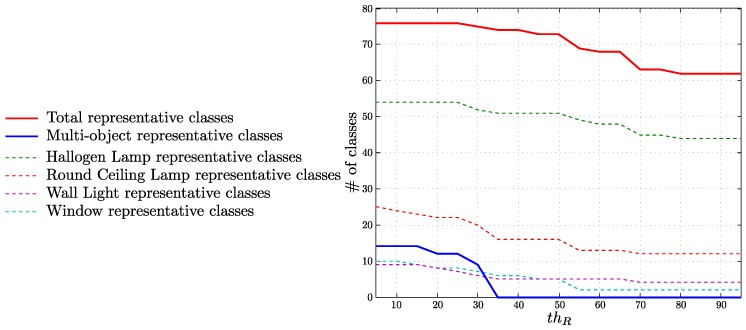
Representative classes for each object and representative classes associated to more than one object (multi-object classes) for different values of $th_{R}$. In the analyzed vocabulary, 342 classes were created.

Figure 8 shows the number of representative classes for each object and the number of representative classes associated to more than one object for different values of $th_{R}$. Even for high values of $th_{R}$, we find representative classes for all the objects. The number of representative classes associated to more than one object is small for low values of $th_{R}$, and null for $th_{R}$ higher than 35%

**Figure 9:**
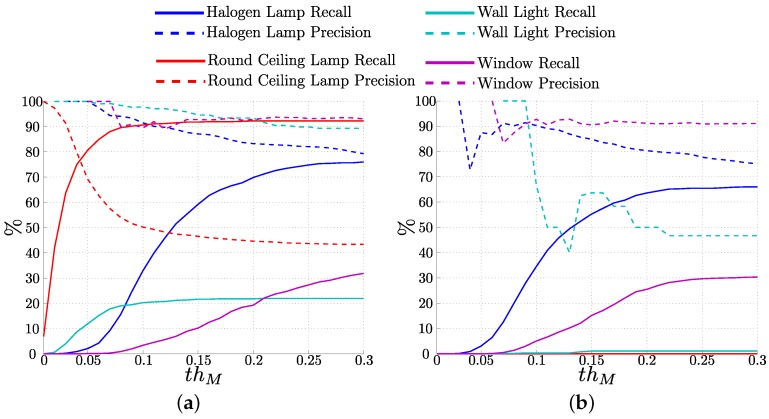
Precision (**dashed line**) and Recall (**solid line**) for the detection of real objects as the matching threshold $th_{M}$ varies. We can see better performance when including altitude in the descriptor, together with SURF appearance (**a**) that using only SURF (**b**).

Next, we want to quantify how new occurrences of objects are recognized using the representative classes. The representative classes of each annotated object are selected in the training data with $th_{R}$ equal 50%. In the test data, key-points detected as being of a representative class of an object are labeled as being generated by that object. Figure 9a shows precision and recall when classifying features into object labels for different values of the similarity threshold, $th_{M}$.

The highest recall, above 90%, was achieved for the detection of Round Ceiling Lamps, which appear in almost all the rooms traversed during the sequence. However, the precision is low for this class, below 50% for higher values of $th_{M}$. For the rest of the objects, precision is above 90%. For Halogen Lamps, recall reaches almost 80%, while for Wall Lights and Windows recall values are lower, about 20% and 30%, respectively. Those objects have similar appearance and the areas of the image where they appear are the same so they are really hard to distinguish.

##### Inclusion of the Altitude Values

Figure 9b shows the same plot that Figure 9a, but, in this case, the altitude has not been used in the process. While the results are similar for Halogen Lamps and Windows, the accuracy for Wall Lights decreases from around 20% to 2% when we do not use the altitude. Even more dramatic is the change in the detection of Round Ceiling Lamps: without the altitude value, no Round Lamps are detected. In our case, the image descriptors of features around the Round Ceiling Lamps are similar to the descriptors created by other entities of the environment, so the appearance descriptor is not enough to distinguish this object.

#### 4.5.3. Qualitative Object-Class Correspondence

**Figure 10:**
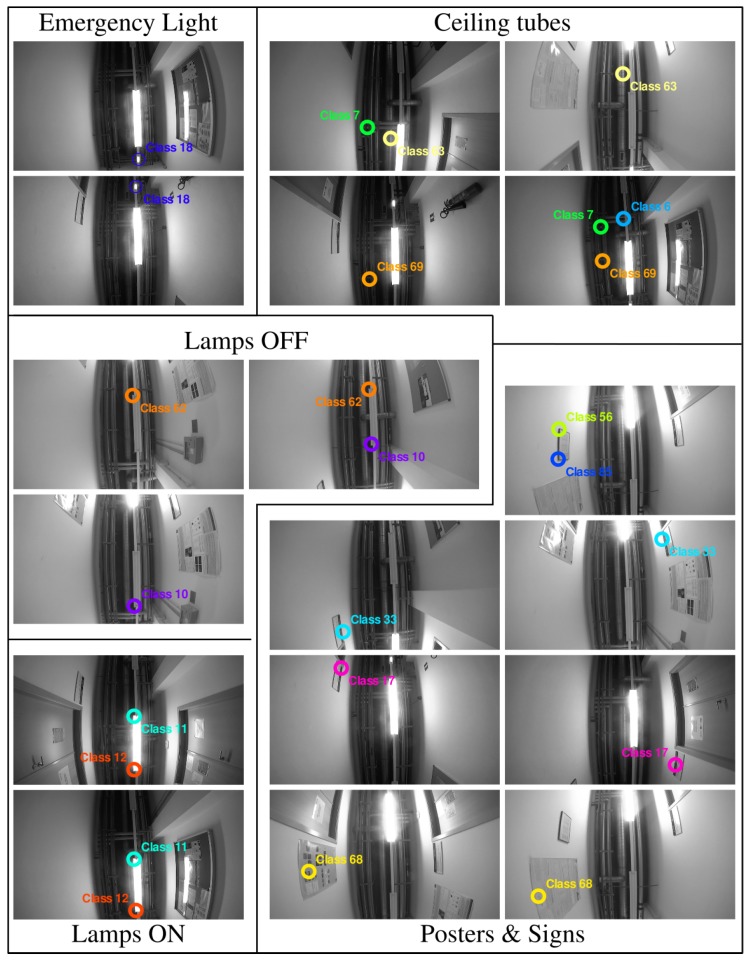
Examples of classes created by our method that correspond to real scene elements. Most of the classes are detected on various frames, and on different sides of the images. (Best seen in color).

Figure 10 shows examples of the correspondence between the classes created by our vocabulary and the elements of the environment. For this experiment, we have run our vocabulary creation method in the second sequence presented. Again, most of the objects detected are lamps, halogen lamps (on and off) and emergency lights. Ceiling tubes, posters and labels are also detected. Occurrences in different frames are shown for most of the classes. Note that classes 33 and 17, which correspond to wall signs, are detected in both sides of the corridor.

## 5. Applications Using the Proposed Vocabulary

This section describes two possible applications of the vocabulary presented in this work. Place recognition and object detection are studied here.

### 5.1. Place Recognition

One of the applications where BoW representations have been widely used is place recognition. The next experiment shows the performance using a vocabulary created with our proposal and compares it with the performance using standard *k*-means vocabulary.

**Figure 11:**
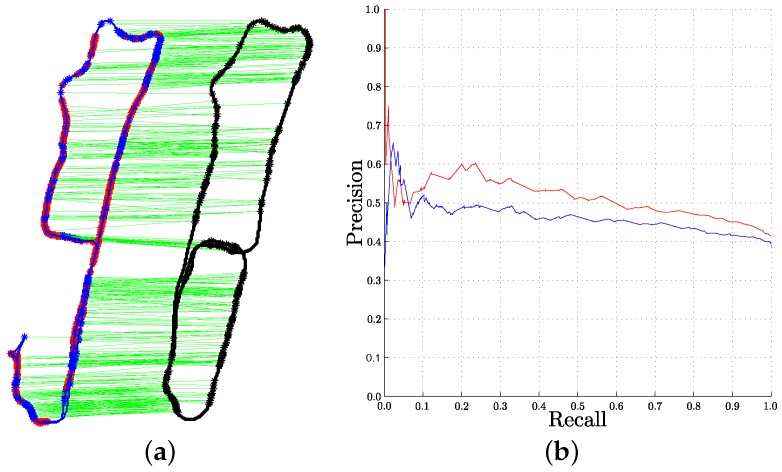
Our vocabulary *vs.* standard *k*-means vocabulary for Place Recognition. (**a**) odometry of the two sequences used: Test (**blue**) and Train (**black**). Correct localization results are shown as green lines, and errors are shown with red points in the test trajectory; (**b**) precision-recall curves using our enhanced vocabulary (**red**) and *k*-means vocabulary (**blue**). These curves have been obtained varying $th_{HistDist}$. (Best seen in color).

The trajectories of the two sequences included in our data-set are shown in Figure 11. The ground truth of the sequences are aligned so the positions in both trajectories can be compared. We obtained this aligned ground truth using the g2o optimization tool [52].

According to BoW representations, each image is represented by a histogram of the number of occurrences of each word in the image. Each histogram is normalized with the total number of words in the corresponding image, and the image similarity is obtained according to these histograms distance. The localization of a test image is considered to be the same as the localization of the most similar training image found. If the most similar training image found is within a similarity distance larger than a threshold ($th_{HistDist}=0.2$ in our case), we consider that the location is unknown or uncertain, therefore no answer is given. The localization will be considered correct if the match and the test odometry positions are within 1.5 m. Figure 11 shows the results of the experiment, including the visualization of the test and train trajectories and the precision-recall curve. We can observe how our approach gives better results than those obtained using a standard *k*-means vocabulary.

**Figure 12:**
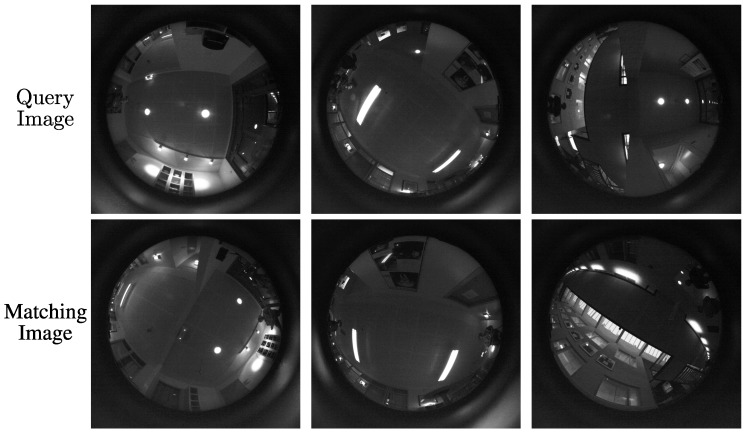
Examples of correct place recognition where the robot location is rotated significantly between test and training images. The left image of each row shows the query test image, and the right image show the correctly matched image from the training data.

Finally, we show some examples of correct place localization when the robot orientation is very different between train and test in Figure 12. In these cases, our model was robust enough to classify those images as being the same location.

### 5.2. Object Detection

Section 4.5 shows how the vocabulary represents some of the objects found in the environment. Figure 3 shows the detection of objects in different frames. For this experiment, we have used the representative classes of each object. Key-points detected as being of a representative class of an object are labeled as being generated by that object. The position of the markers, green circles for correct detections and red crosses for incorrect ones, correspond to the position where the key-points where detected in the image. In those examples, we can see how most of the Round Ceiling Lamps are correctly detected. In the first image of the bottom row, the red arrow shows points classified as Round Ceiling Lamp that correspond to a different kind of lamp that newly appears in this room. We can also see how the areas labelled as windows are very dissimilar due to the objects seen through these windows and their different shapes.

## 6. Conclusions

This work presents a new method to create an enhanced visual vocabulary from an image sequence that learns semantic relationships between visual words of the working environment. The key elements of the method are the use of tracked scene points and the inclusion of information of the key-points altitude when building the visual words. Our approach is focused on long term indoor robotics monitoring applications. With this purpose, we consider systems where the camera points to the ceiling, which facilitates the acquisition of more stable and repetitive scene elements. The experimental validation, with indoor sequences acquired from a mobile robotic platform, shows the performance and the enhanced semantic properties for different method parameters. Comparisons with the standard *k*-means vocabulary present our method as a richer alternative for the usual BoW approach to build a visual vocabulary. Our method provides more representative semantic information of the environment, including relationships between visual words and environment objects or parts. At the same time, our method has shown better results in a place recognition application than the usual BoW approach, and promising results for object discovery and semantic representation for long term place recognition in a robot operating environment.

## Figures and Tables

**Table 1:** Normalized entropy of the object classification into classes or words.

**Words**			
**Object**	**Our Approach DBSCAN**	**Our Approach Hierarchical Clustering**	**Standard *k*-Means**
Halogen Lamp	0.598	0.598	0.599
Round Ceiling Lamp	0.454	0.454	0.450
Wall Light	0.434	0.439	0.424
Window	0.441	0.450	0.441
**Mean**	0.482	0.485	0.479
**Classes**			
**Object**	**Our Approach DBSCAN**	**Our Approach Hierarchical Clustering**	**Standard *k*-Means**
Halogen Lamp	0.460	0.524	−
Round Ceiling Lamp	0.356	0.497	−
Wall Light	0.840	0.266	−
Window	0.112	0.290	−
**Mean**	0.275	0.394	−
